# Dietary Supplementation with Chenodeoxycholic Acid or Ursodeoxycholic Acid Modulates Growth, Thyroid Status, and Hepatopancreatic–Intestinal Health in Juvenile Little Yellow Croaker *Larimichthys polyactis*

**DOI:** 10.3390/antiox14111325

**Published:** 2025-11-03

**Authors:** Rui Wu, Limin Yan, Yao Li, Ting Ye, Yu Zhang, Wei Zhan, Chenglong Wu, Bao Lou, Xiao Liang

**Affiliations:** 1 National-Local Joint Engineering Laboratory of Aquatic Animal Genetic Breeding and Nutrition (Zhejiang), Huzhou University, 759 East 2nd Road, Huzhou 313000, China; wurui6666@outlook.com (R.W.); 18338319753@163.com (Y.L.); 01998@zjhu.edu.cn (C.W.); 2 State Key Laboratory for Quality and Safety of Agro-Products, Institute of Hydrobiology, Zhejiang Academy of Agricultural Sciences, Desheng Middle Road 298, Hangzhou 310021, China; 13197982570@163.com (L.Y.); yet@zaas.ac.cn (T.Y.); zhangy@zaas.ac.cn (Y.Z.); zhanwei@zaas.ac.cn (W.Z.); 3 College of Fisheries and Life Science, Dalian Ocean University, Dalian 116023, China

**Keywords:** chenodeoxycholic acid, ursodeoxycholic acid, thyroid, hepatic–intestinal health, *Larimichthys polyactis*

## Abstract

Commercial feeds formulated for *Larimichthys crocea* are commonly used in intensive farming of *Larimichthys polyactis*; however, their nutritional composition is suboptimal for the latter. The study evaluated the effects of dietary chenodeoxycholic acid (CDCA) and ursodeoxycholic acid (UDCA) supplementation on mitigating nutritional mismatch-induced growth retardation and hepatopancreatic–intestinal metabolic disorders in *L. polyactis*. Fish were fed seven feeds: a commercial feed (control) and feeds supplemented with 300, 600, and 1200 mg/kg of CDCA or UDCA. Results showed that both bile acids (BAs) supplementation improved growth, altered thyroid hormone metabolism, with significant changes in hepatopancreatic–intestinal types of deiodination. Both BAs increased hepatopancreatic energy metabolism and cholic acid synthesis, while inducing hepatopancreatic oxidative damage. Notably, 300 mg/kg CDCA and 600 mg/kg UDCA significantly reduced hepatopancreatic vacuolation and lipid accumulation, which was associated with enhanced protease and lipase activities (*p* < 0.05). Dietary both BAs supplementation enhanced intestinal antioxidant capacity, but contributed to the inflammation, with 300 mg/kg UDCA improving intestinal mucosal morphology (*p* < 0.05). These findings suggest that supplementation with dietary 300 mg/kg CDCA, 300 and 600 mg/kg UDCA could alleviate growth restriction and liver–intestinal structural damage caused by nutritional mismatch, reduce hepatic fat accumulation, and enhance intestinal antioxidant capacity of *L. polyactis*.

## 1. Introduction

The little yellow croaker (*Larimichthys polyactis*), an economically significant species in mariculture, is widely distributed along the coastal regions of southeastern China [1]. Currently, *L. polyactis* is well-suited for full-scale artificial aquaculture, but its nutritional requirements across various growth stages remain insufficiently studied [2]. Given its close genetic relationship with the large yellow croaker (*Larimichthys crocea*) [3], commercial diets formulated for the *L. crocea* are commonly used in the artificial farming of the *L. polyactis*. Research has shown that imbalances in feed composition or the presence of antinutritional factors can negatively affect the health of the liver and intestine, rendering the fish more susceptible to diseases [4]. Moreover, inappropriate nutrient levels can impair the digestive system of fish [5]. Previous studies have demonstrated that a dietary protein-to-lipid ratio of 47:12 is optimal for juvenile *L. polyactis*, and significant differences have been observed in the primary nutritional requirements between *L. crocea* and *L. polyactis* [2,6,7]. This disparity may account for the vacuolation and lipid accumulation in hepatopancreatic cells, as well as the intestinal inflammation observed in *L. polyactis* when fed commercial diets formulated for *L. crocea*. The antinutritional factors, inappropriate content of nutrients, and new compounds can impair liver and gut health, in turn, exacerbate stress responses, suppress immune function, and limit growth performance [5]. These factors directly influence the farming efficiency of *L. polyactis* under intensive aquaculture conditions. Several studies have confirmed that exogenous supplementation of bile acids (BAs), such as chenodeoxycholic acid (CDCA), ursodeoxycholic acid (UDCA), and taurocholic acid (TCA), can effectively regulate tissue energy metabolism and enhance the overall health of fish [8,9]. Consequently, modulating liver and gut metabolic functions through dietary BAs may offer a promising strategy to mitigate dysfunction and improve the health status of *L. polyactis* in aquaculture.

BAs serve as vital functional feed additives that can significantly enhance nutrient absorption, reduce adiposity, and mitigate metabolic stress in fish [9]. In recent years, BAs have garnered recognition as key signaling molecules, with the enterohepatic circulation of BAs playing a pivotal role in the maintenance of metabolic homeostasis [10,11]. The hepatic biosynthesis of BAs begins with cholesterol as the precursor, undergoing oxidative modifications catalyzed by CYP450 enzymes, specifically through the CYP7A1 and CYP27A1 pathways, resulting in the production of primary BAs, including cholic acid (CA) and CDCA [8]. These primary BAs are subsequently released into the intestinal lumen, where they are dehydroxylated and deconjugated by the intestinal microbiota, leading to the formation of secondary BAs [12,13]. The circulation of BAs is primarily regulated by the liver-intestine axis, with key receptors, including the farnesoid X receptor (FXR) and G protein-coupled bile acid receptor 1 (TGR5), playing integral roles in this process [11]. Notably, various BAs exhibit distinct activation profiles toward their respective receptors, and the species and concentrations of exogenous BAs can significantly influence homeostatic regulation [8]. Noteworthy compounds, such as CDCA, TCA, taurodeoxycholic acid (TDCA), UDCA, and lithocholic acid (LCA), have been extensively investigated in numerous fish species [9].

Previous studies have demonstrated the potential benefits of CDCA and UDCA supplementation in aquafeeds, particularly for enhancing growth, maintaining the integrity of the intestinal structure, and reducing hepatic apoptosis and lipid accumulation in fish [8,9]. In *L. crocea*, supplementation with 300 and 900 mg/kg CDCA in a high soybean oil diet resulted in significant growth improvements [14]. However, when incorporated into a high-lipid diet, the same dosages did not affect growth but enhanced liver total lipase activity [15,16]. In contrast, supplementation with 300, 600, and 900 mg/kg CDCA in high-lipid diet did not significantly affect growth or liver lipase activity, but these dietary supplementations were found to improved intestinal fold height in largemouth bass (*Micropterus salmoides*) [17]. Moreover, CDCA has been shown to activate FXR and exert anti-inflammatory effects by directly and indirectly suppressing the NF-κB pathway in *L. crocea* [18]. In Japanese flounder (*Paralichthys olivaceus*), supplementation with 250 mg/kg UDCA in a normal diet increased liver total lipase activity but had no significant effect on growth [19]. In juvenile *L. crocea*, feeding diets containing 50 and 100 mg/kg UDCA resulted in improved growth performance, enhanced lipid metabolism, and a reduction in pro-inflammatory signaling [20,21]. These findings suggest that both CDCA and UDCA play a role in modulating enterohepatic function and growth performance in fish. However, their effects appear to be species-specific and influenced by dietary composition.

To mitigate the adverse effects associated with inadequate nutrient intake, the present study aims to investigate the effects of dietary CDCA or UDCA supplementation on growth, thyroid status, and hepatopancreatic–intestinal function in *L. polyactis*, when fed a commercial diet formulated for *L. crocea*. The results may identify potential nutritional strategies to alleviate liver and gut dysfunction in fish, ultimately enhancing aquaculture productivity.

## 2. Materials and Methods

### 2.1. Animal Ethics

The fish experiments were conducted in compliance with the “Guidelines for Experimental Animals” issued by the Ministry of Science and Technology (Beijing, China), and were approved by the Committee on Laboratory Animal Experimentation at the Zhejiang Academy of Agricultural Sciences (Permit Number: 2023ZAASLA29). All researchers involved in the experiments underwent appropriate training and received individual authorization.

### 2.2. Diets

The commercial diet used in this study was sourced from Fuzhou Haima Feed Co., Ltd., Fuzhou 350002, China. The nutrient composition of the experimental diets is detailed in Table 1. In accordance with previous studies [14,22], three levels of BA supplementation (300 mg/kg, 600 mg/kg, and 1200 mg/kg) were incorporated into the basal diet. The diet formulation adhered to the method outlined in a prior study [23]. The diet was uniformly milled, followed by the incorporation of either CDCA or UDCA (Macklin, Shanghai, China) with a purity of 99%, at the specified concentrations. The mixtures were subsequently homogenized and re-pelletized using a standard food processor. After processing, the samples were dried at 30 °C for 24 h and stored at −20 °C.

### 2.3. Fish-Feeding Experiment

*L. polyactis* juveniles were procured from the Xiangshan Harbor Aquatic Seedling Co. Ltd. (Xiangshan County, Ningbo, China). The experiment was conducted at the Zhoushan Fisheries Research Institute (Zhoushan, China). In this experiment, the same commercial feed was used during the acclimation period and the feeding trial. Prior to the commencement of feeding trial, the juveniles were acclimated in indoor cage tanks for a period of two weeks and fed the Con diet. After the acclimation period, the juveniles were fasted for 24 h and subsequently weighed. Healthy juveniles (initial mean weight 7.34 ± 0.23 g) were then randomly distributed into 21 laboratory tanks (200 L), with 30 individuals per tank. Each diet was assigned to triplicate tanks. The fish were hand-fed twice daily to apparent satiation at 05:00 and 17:00, and the daily feed consumption in each tank was meticulously recorded. The feeding trial spanned 8 weeks. Throughout the experimental period, the water temperature fluctuated between 23.5 and 27.2 °C, salinity ranged from 27.2‰ to 29.7‰, and dissolved oxygen levels were maintained at or above 8.0 mg/L.

### 2.4. Sample Collection

Following the feeding experiment, all fish from each tank were anesthetized with 0.06 g/L MS-222 (Sigma Diagnostics INS, St. Louis, MO) and weighed after a 24 h fasting period. Concurrently, body length and feed consumption data were recorded. A total of 45 fish from each group (15 fish per tank) were randomly selected for the collection of serum, hepatopancreas, and anterior intestine samples for subsequent analysis. Blood samples (5 fish in each replicate) were collected from the caudal vein of all treatments from anesthetized fish. Blood sample was centrifuged at 1500× *g* for 30 min at 4 °C, and then the non-hemolyzed serum was gathered using a micro-pipette and stored at −80 °C until use. The hepatopancreas from 9 fish per tank were homogenized in liquid nitrogen and pooled into a single tube, with a stochastic mixture of 9 fish per tank constituting one replicate. The same procedure as described above was applied for the collection of the anterior intestine. These samples were stored at −80 °C for subsequent gene expression and biochemical parameter analyses. Additionally, the hepatopancreas and anterior intestine from 3 fish per group were harvested for histological examination.

### 2.5. Biochemical Parameters Analysis

#### 2.5.1. Serum Biochemical Analysis

Serum concentrations of total triiodothyronine (T3), thyroxine (T4), free triiodothyronine (FT3), free thyroxine (FT4), thyroid-stimulating hormone (TSH), aspartate transaminase (AST), alanine transaminase (ALT), and AKP (alkaline phosphatase) were measured using enzyme-linked immunosorbent assay (ELISA) kits. All assays were performed according to the manufacturer’s protocols, using commercial kits obtained from Shanghai Enzyme-linked Biotechnology Co., Ltd. (Shanghai, China). The T3/T4 ratios were calculated according to the results of T3 and T4 levels.

#### 2.5.2. Biochemical Indicators and Enzyme Activity in Hepatopancreas and Intestine

The activities of deiodinases (DIOs), total superoxide dismutase (T-SOD), catalase (CAT), total antioxidant capacity (T-AOC), glutathione (GSH), and malonaldehyde (MDA) were measured in both the hepatopancreas and intestine. Additionally, levels of interleukin-8 (IL-8), interleukin-1β (IL-1β), tumor necrosis factor-α (TNF-α), total protease, total lipase, and total amylase were quantified in the intestine. Total lipase activity in the hepatopancreas was also determined. All assays were conducted following the instructions provided by the commercial kits from Shanghai Enzyme-linked Biotechnology Co., Ltd. (Shanghai, China). The key parameters were summarized in Table 2.

### 2.6. Histochemical and Histological Examination

Histological and histochemical examinations were conducted as described by Li et al. [24]. Hematoxylin and eosin (H&E) staining, along with Oil Red O staining, were performed to assess the histology of the hepatopancreas and intestine. For histological analysis, tissue sections from the same region of the hepatopancreas or anterior intestine of three fish per group were fixed in 4% paraformaldehyde for 24 h, followed by transfer to 70% ethanol for preservation. The samples were then dehydrated through a graded series of ethanol concentrations, treated with xylene to remove the ethanol, and embedded in paraffin wax. Sections (5 μm thick) were cut using a rotary microtome (HM 340E, Thermo Scientific™, Waltham, MA, USA) and stained with hematoxylin and eosin. The slides were mounted with DPX mounting medium. For histochemical analysis, samples from the same region of the hepatopancreas were sectioned at 6 μm using a cryostat microtome, fixed in cold 10% buffered formalin, and stained with Oil Red O. Images were captured using a microscope with a camera (Leica DM3000 LED, Wetzlar, Germany) at 400× magnification.

### 2.7. Total RNA Extraction and qRT-PCR Analysis

Total RNA was isolated from the hepatopancreas and intestine of fish from seven dietary groups using RNA isoPlus (TaKaRa, Shiga, Japan). cDNA synthesis for subsequent quantification was performed using Hifair™ II 1st Strand cDNA Synthesis SuperMix for qPCR (gDNA digester plus) (Yeasen, Shanghai, China). A total of 1 µg of RNA from each sample was used to synthesize the first-strand cDNA. The expression levels of TH and BA metabolism genes in the hepatopancreas and intestine were assessed in response to various diets. Quantitative real-time PCR (qRT-PCR) was carried out using the Quant Studio 6 Flex Real-Time PCR System (Life Technologies, Beijing, China), following the manufacturer’s instructions for Hieff™ qPCR SYBR Green Master Mix (Low Rox Plus) (2X) (Yeasen, Shanghai, China). Relative mRNA expression values were determined using the comparative Ct (2^−ΔΔCt^) method. *18S rRNA* was employed as the internal reference gene. The target genes and their corresponding mRNA sequences were retrieved from the genomes and intestinal transcriptome of *L. polyactis* [3]. The specific primers for the target genes and reference gene are provided in Table 3.

### 2.8. Calculations and Statistical Analyses

The parameters were calculated as follows:

Weight gain rate (WGR, %) = 100 × (final body weight − initial body weight)/initial body weight)

Specific growth rate (SGR, % day^−1^) =100 × (Ln final body weight − Ln initial body weight)/t

Feed conversion rate (FCR) =100 × (final body weight − initial body weight)/feed consumed (g)

Condition Factor (CF, g/cm^3^) = 100 × (final body weight)/(final body weight)^3^

Statistical analyses were performed using SPSS 22.0 (IBM, New York, NY, USA). Before analysis, all data were verified for normality and homoscedasticity of variance by Shapiro–Wilk’s and Levene’s test, respectively. The data were analyzed using one-way ANOVA and followed by Tukey’s multiple comparison. A significance level of *p* < 0.05 was considered statistically significant. When data did not have a homogeneous variation, the non-parameter Kruskal–Wallis test was applied, and followed by all pairwise multiple comparisons if the results of Kruskal–Wallis test showed significant difference (*p* < 0.05). All data are presented as mean ± standard error (SE). Correlations among the variables were evaluated using the Mantel test via the OmicShare tools, a free online platform for data analysis (http://www.omicshare.com/tools, accessed on 30 April 2024).

## 3. Results

### 3.1. Growth Performance, Feed Utilization and Morphological Indices

The growth performance of *L. polyactis* fed different diets is summarized in Table 4. The indexes of WGR and SGR in the CDCA and UDCA groups were significantly higher compared to the Con group (*p* < 0.05). No significant differences in CF were observed among the fish fed the various diets (*p* > 0.05). Furthermore, the FCR was significantly lower in the CDCA 600 group (*p* < 0.05), whereas no significant differences in FCR were found between the Con and UDCA groups (*p* > 0.05).

**Figure A1:**
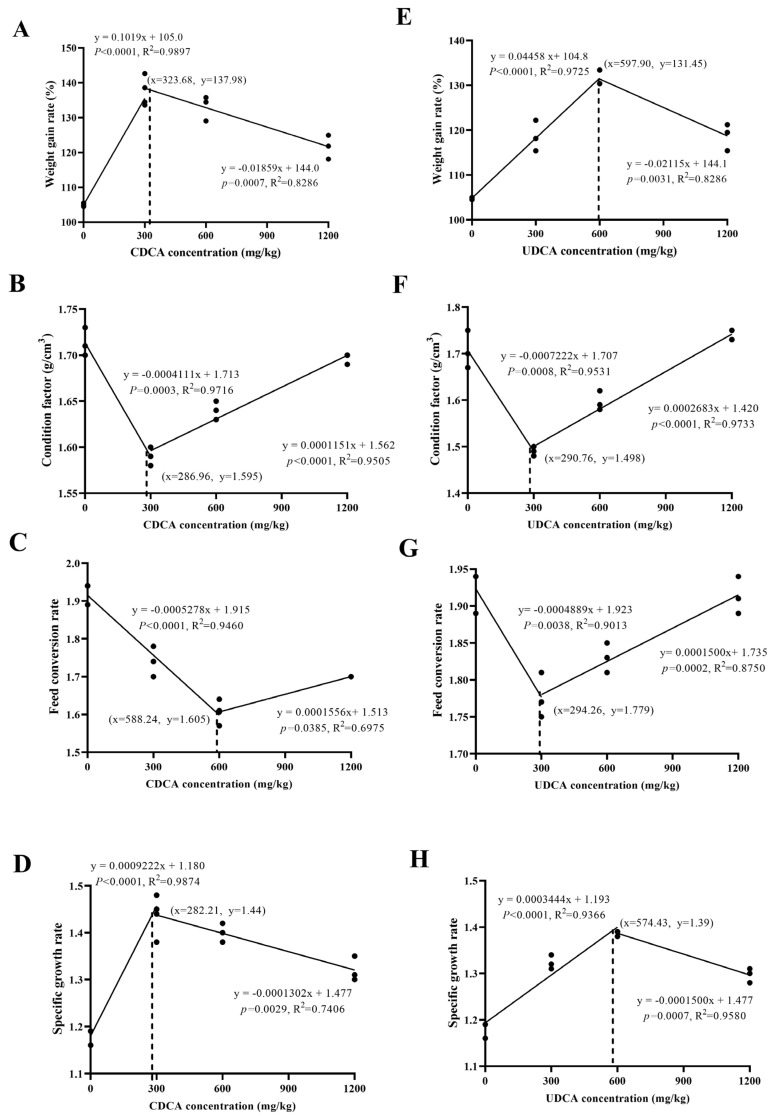
Relationship between different dietary BA supplementation and growth indices.

The relationships between different dietary BA supplementation and growth indices are illustrated in Figure A1. The WGR, CF, SGR, and FCR are best described by a broken-line model. Based on the graphical analysis, the optimal CDCA supplementation concentrations for achieving the highest WGR (137.98%) and SGR (1.44) were 323.68 mg/kg and 282.21 mg/kg, respectively. The optimal UDCA supplementation concentrations for achieving the highest WGR (131.45%) and SGR (1.39) were 597.90 mg/kg and 574.43 mg/kg (Appendix A, Figure A1(A,D,E,H)). The optimal CDCA supplementation concentrations for the lowest CF (1.595) and FCR (1.605) were 286.96 mg/kg and 588.24 mg/kg, while the optimal UDCA supplementation concentrations for the lowest CF (1.498) and FCR (1.779) were 290.76 mg/kg and 294.26 mg/kg (Appendix A, Figure A1(B,C,F,G))

### 3.2. Digestive Enzymes

**Figure 1:**
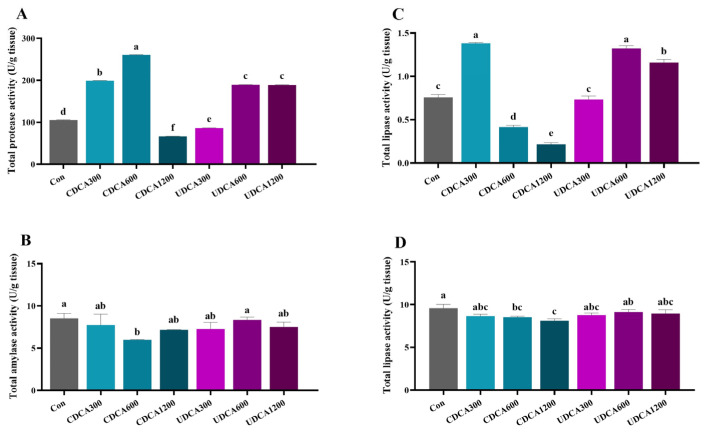
Effects of dietary BA supplementation on digestive enzyme activities in *L. polyactis*. (**A**) intestinal protease activity; (**B**) intestinal amylase activity; (**C**) intestinal lipase activity; (**D**) liver lipase. The values were expressed as mean ± SE (*n* = 3), and different little letters indicate significant differences among all the groups (*p* < 0.05).

In the intestine, total protease activity in the CDCA300, CDCA600, UDCA600, and UDCA1200 groups was significantly higher compared to the Con group, whereas activity in the CDCA1200 and UDCA300 groups was significantly lower (*p* < 0.05) (Figure 1A). Total amylase activity in the CDCA600 group was significantly reduced relative to the Con group (*p* < 0.05) (Figure 1B). Total lipase activity in the CDCA300, UDCA600, and UDCA1200 groups was significantly elevated compared to the Con group, while activity in the CDCA600 and CDCA1200 groups was significantly reduced (*p* < 0.05) (Figure 1C). In the hepatopancreas, total lipase activity in the CDCA600 and CDCA1200 groups was significantly lower than that in the Con group (*p* < 0.05) (Figure 1D).

### 3.3. Serum THs Levels

**Figure 2:**
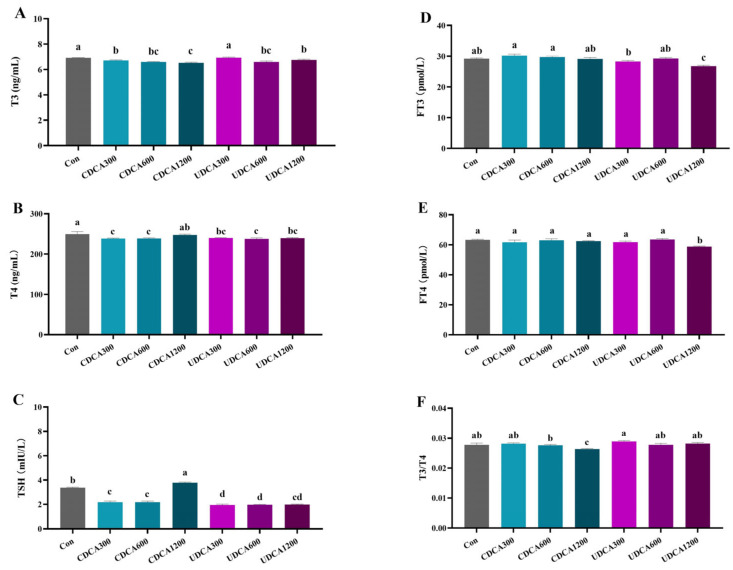
Effects of dietary BA supplementation on serum TH levels in *L. polyactis*. (**A**) T3; (**B**) T4; (**C**) TSH; (**D**) FT3; (**E**) FT4; (**F**) T3/T4. The values were expressed as mean ± SE (*n* = 3), and different little letters indicate significant differences among all the groups (*p* < 0.05).

The serum THs levels were significantly altered by various BA treatments. Supplementation with dietary CDCA at doses ranging from 300 to 1200 mg/kg, as well as dietary UDCA at doses ranging from 600 to 1200 mg/kg, both resulted in a significant decrease in T3 levels (*p* < 0.05) (Figure 2A). A significant reduction in T4 and TSH levels was observed in the CDCA300, CDCA600, and all UDCA treatment groups (*p* < 0.05) (Figure 2B,C). Furthermore, FT3 and FT4 levels were only significantly reduced in the UDCA1200 group (Figure 2D,E) (*p* < 0.05). Notably, TSH levels were significantly elevated in the CDCA1200 group, while the ratio of T3/T4 was significantly decreased in the same group (Figure 2F) (*p* < 0.05).

### 3.4. DIO Activity

**Figure 3:**
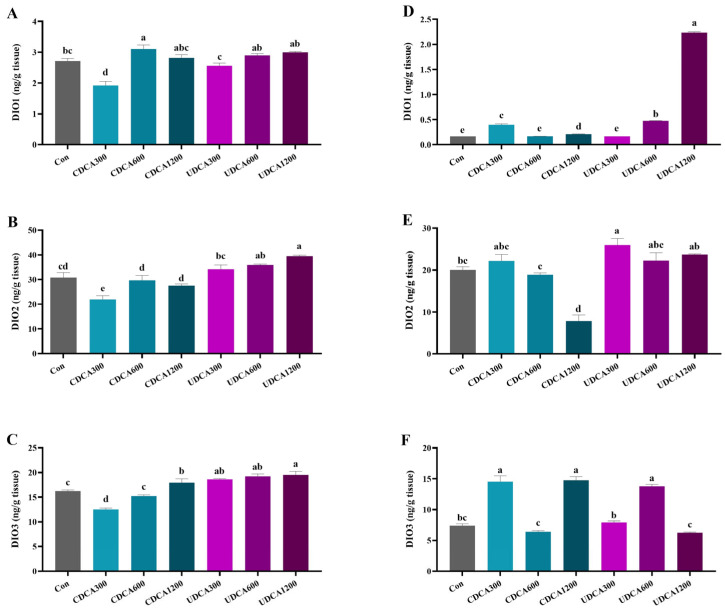
Effects of dietary BA supplementation on serum TH levels in *L. polyactis*. (**A**) hepatopancreatic DIO1 activity; (**B**) hepatopancreatic DIO2 activity; (**C**) hepatopancreatic DIO3 activity; (**D**) intestinal DIO1 activity; (**E**) intestinal DIO2 activity; (**F**) intestinal DIO3 activity. The values were expressed as mean ± SE (*n* = 3), and different little letters indicate significant differences among all the groups (*p* < 0.05).

The activities of hepatopancreatic DIOs in the CDCA300 group were significantly lower than those in the Con group, while the activities of hepatopancreatic DIO1 in the CDCA600 group and DIO3 in the CDCA1200 group were significantly higher than those in the Con group (*p* < 0.05) (Figure 3A–C). No significant differences were observed in the activity of hepatopancreatic DIO1 between the UDCA treatment groups and the Con group (*p* > 0.05) (Figure 3A). The activity of hepatopancreatic DIO3 in the UDCA300 group was significantly elevated compared to the Con group, and both DIO2 and DIO3 activities in the UDCA600 and UDCA1200 groups were significantly increased compared to the Con group (*p* < 0.05) (Figure 3B,C).

The activities of intestinal DIO1 and DIO3 in the CDCA300 and CDCA1200 groups were significantly higher compared to the Con group (Figure 3D,F). Conversely, the activity of intestinal DIO2 in the CDCA1200 group was significantly lower than that in the Con group (*p* < 0.05) (Figure 3E). The activity of intestinal DIO2 in the UDCA300 group was significantly higher than that in the Con group (*p* < 0.05) (Figure 3E). Additionally, the activities of intestinal DIO1 and DIO3 in the CDCA600 group were significantly higher than those in the Con group, and the activity of intestinal DIO3 in the UDCA1200 group was also significantly increased compared to the Con group (*p* < 0.05) (Figure 3D,F).

### 3.5. Hepatopancreatic and Intestinal Morphology

**Figure 4:**
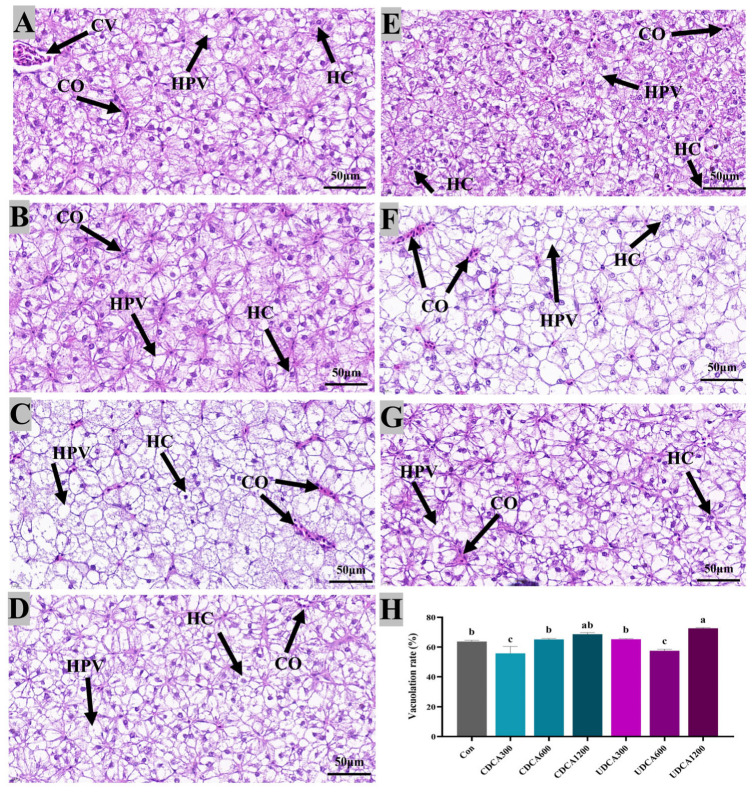
Effects of dietary BA supplementation on H&E-stained sections (80×) of hepatopancreas in *L. polyactis*. The morphology was observed under microscope, and scale bars indicated 50 μm. (**A**–**C**) H&E-stained sections of hepatopancreas in the CDCA300, 600, and 1200 mg/kg groups; (**D**–**F**) H&E-stained sections of hepatopancreas in the UDCA300, 600, and 1200 mg/kg groups; (**G**) H&E-stained sections of hepatopancreas in the control group; (**H**) hepatopancreatic vacuolation rate. The central venous area, hepatocyte, hepatocyte vacuolation, and congestion were labeled as “CV”, “HC”, “HPV”, and “CO”, respectively. The values were expressed as mean ± SE (*n* = 3), and different little letters indicate significant differences among all the groups (*p* < 0.05).

**Figure 5:**
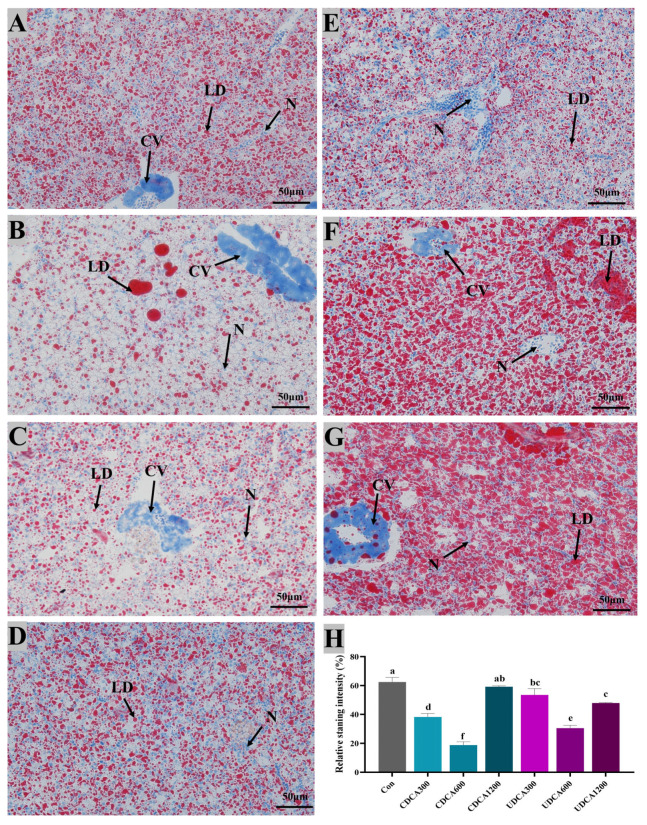
Effects of dietary BA supplementation on Oil-Red-O-stained sections (200×) of hepatopancreas in *L. polyactis*. The morphology was observed under microscope, and scale bars indicated 50 μm. (**A**–**C**) Oil-Red-O-stained sections of hepatopancreas in the CDCA300, 600, and 1200 mg/kg groups; (**D**–**F**) Oil-Red-O-stained sections of hepatopancreas in the UDCA300, 600, and 1200 mg/kg groups; (**G**) Oil-Red-O-stained sections of hepatopancreas in the control group; (**H**) lipid accumulation was quantified by measuring the intensity of the stained oil droplets. Central venous, lipid droplets, and nuclei are marked by “CV”, “LD”, and “N”, respectively. The values were expressed as mean ± SE (*n* = 3), and different little letters indicate significant differences among all the groups (*p* < 0.05).

To assess hepatopancreatic morphology and lipid accumulation, H&E and Oil Red O staining were conducted, and the results were presented in Figure 4 and Figure 5. In all treatment groups, hepatocytes exhibited swelling and vacuolation (Figure 4). The vacuolation rate of hepatocytes in the Con group was significantly higher than that observed in CDCA300 and UDCA600 groups, while it was significantly lower than that in the UDCA1200 group (*p* < 0.05). Furthermore, lipid droplets in the CDCA300, CDCA600, and UDCA-treated groups were significantly reduced compared to those in the Con group (*p* < 0.05). However, no significant differences in the intensity of Oil Red O-stained lipid droplets were found between the CDCA1200 and Con groups (*p* > 0.05).

**Figure 6:**
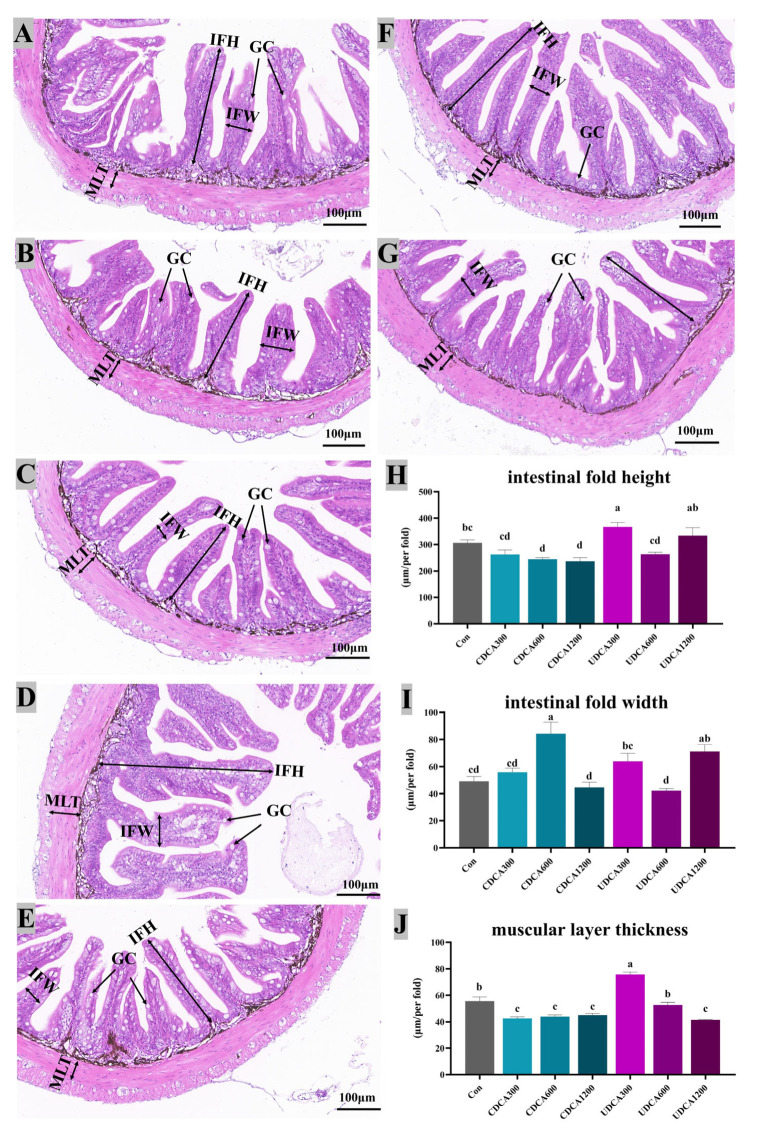
Effects of dietary BA supplementation on intestinal H&E-stained sections (20×) in *L. polyactis*. The morphology was observed under microscope, and scale bars indicated 100 μm. (**A**–**C**) H&E-stained sections of intestine in the CDCA300, 600, and 1200 mg/kg groups; (**D**–**F**) H&E-stained sections of intestine in the UDCA300, 600, and 1200 mg/kg groups; (**G**) H&E-stained sections of intestine in the control group; (**H**) intestinal fold height (IFH); (**I**) intestinal fold width (IFW); (**J**) muscular layer thickness (MLT); GC: Goblet cells. The values were expressed as mean ± SE (*n* = 3), and different little letters indicate significant differences among all the groups (*p* < 0.05).

Intestinal morphology was shown in Figure 6. The results revealed that intestinal fold heights in the CDCA600 and CDCA1200 groups were significantly lower than those in the Con group, while the intestinal fold height in the UDCA300 group was significantly higher than that in the Con group (*p* < 0.05). Additionally, intestinal fold widths in the CDCA600 and UDCA1200 groups were significantly greater than those in the Con group (*p* < 0.05). Mucosa layer thickness in the Con group was significantly greater than that in the CDCA-treated and UDCA1200 groups, but significantly lower than that in the UDCA300 group (*p* < 0.05). No significant differences were observed in the mucosal layer length between the UDCA600 and Con groups (*p* > 0.05).

### 3.6. Hepatopancreatic Antioxidant Parameters and Injury Biomarkers

The antioxidant parameters in the hepatopancreas of *L. polyactis* were significantly affected by the different dietary treatments (Table 5). Compared to the Con group, T-AOC activity was significantly decreased in the CDCA600, UDCA600, and UDCA1200 groups, whereas T-AOC activity was significantly increased in the CDCA300 group (*p* < 0.05). T-SOD activity in the CDCA300 group was significantly higher than that in the Con group, while activity in the other CDCA treatment groups and all UDCA treatment groups was significantly lower than that in the Con group (*p* < 0.05). CAT activity in the CDCA600 and CDCA1200 groups was significantly lower than that in the Con group, while the UDCA1200 treatment group exhibited significantly higher CAT activity compared to the Con group (*p* < 0.05). MDA content was significantly higher in all CDCA and UDCA treatment groups compared to the Con group (*p* < 0.05). GSH content was significantly reduced in the CDCA300 and CDCA600 groups compared with the Con group, while it was significantly increased in the UDCA600 and UDCA1200 groups (*p* < 0.05).

Furthermore, ALP activity in the CDCA600 and UDCA300 groups was significantly higher than that in the Con group (*p* < 0.05) (Table 6). ALT activity in the CDCA300, CDCA600, and UDCA1200 groups was significantly higher than those in the Con group, whereas ALT activity in the UDCA300 group was significantly lower than that in the Con group (*p* < 0.05). AST activity in all CDCA treatment groups and the UDCA300 group was significantly lower than that in the Con group, while AST activity in the UDCA1200 group was significantly higher than that in the Con group (*p* < 0.05).

### 3.7. Intestinal Antioxidant Parameters and Pro-Inflammatory Cytokines Markers

The antioxidant parameters in the intestine of *L. polyactis* were significantly influenced by different dietary treatments (Table 7). Compared with the Con group, T-AOC activity and MDA content were significantly reduced in all CDCA and UDCA treatment groups (*p* < 0.05). T-SOD activity in the CDCA600, CDCA1200, UDCA300, and UDCA1200 groups was significantly higher than that in the Con group (*p* < 0.05). CAT activity was significantly elevated in all CDCA treatment groups and in the UDCA300 and UDCA1200 groups compared to the Con group, whereas the UDCA600 group exhibited significantly lower CAT activity (*p* < 0.05). GSH content was significantly lower in the CDCA300, CDCA1200, and all UDCA treatment groups compared to the Con group, while GSH content in the CDCA600 group was significantly higher (*p* < 0.05). Among the CDCA and UDCA treatment groups, the 600 mg/kg concentration exhibited higher T-AOC and GSH levels and lower CAT and MDA levels compared to the 300 mg/kg and 1200 mg/kg treatment groups.

Furthermore, the levels of IL-8, IL-1β, and TNF-α were significantly elevated in all CDCA and UDCA treatment groups compared to the Con group (*p* < 0.05) (Table 8).

### 3.8. Expression of Genes Related to Energy and BA Metabolism

**Figure 7:**
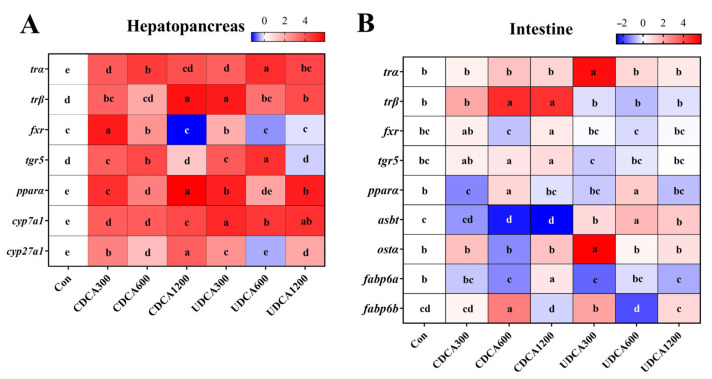
Effects of dietary BA supplementation on the relative expression levels of genes related to energy and BA metabolism in *L. polyactis*. (**A**) Genes in the hepatopancreas; (**B**) genes in the intestine. The data (*n* = 3) were normalized by log2FC, and the results are presented as heatmaps. The red and blue colors indicate the genes whose expression was up-regulated and down-regulated, respectively. Different letters represent significant differences among different treatments in the same tissue (*p* < 0.05).

The expression levels of hepatopancreatic TH and BA metabolism-related genes in *L. polyactis* were significantly influenced by the different dietary treatments (Figure 7A). The expression levels of *trα* and *cyp7a1* in the Con group were significantly lower than those in all CDCA and UDCA treatment groups (*p* < 0.05). The expression levels of *trβ* in the Con group were significantly lower than those in all CDCA and UDCA treatment groups, except for the CDCA600 group (*p* < 0.05). The expression levels of *fxr* in the Con group were significantly lower than those in the CDCA300, CDCA600, and UDCA300 groups (*p* < 0.05). Moreover, the expression levels of *tgr5* in the Con group were significantly lower than those in the CDCA300, CDCA600, UDCA300, and UDCA600 groups (*p* < 0.05). The expression levels of *pparα* and *cyp27a1* in the Con group were significantly lower than those in all CDCA and UDCA treatment groups, except for the UDCA600 group (*p* < 0.05).

The expression levels of intestinal TH and BA metabolism-related genes in *L. polyactis* were also significantly altered by the different dietary treatments (Figure 7B). The expression levels of *trα* and *ostα* in the Con group were significantly lower than those in the UDCA300 groups (*p* < 0.05). The expression levels of *trβ* and *tgr5* in the Con group were significantly lower than those in the CDCA600 and CDCA1200 groups (*p* < 0.05). The expression levels of *fxr* in the Con group were significantly lower than those in the CDCA1200 group (*p* < 0.05). Moreover, the expression levels of *pparα* in the Con group were significantly lower than those in the CDCA600 and UDCA600 groups, but significantly higher than those in the CDCA300 group (*p* < 0.05). The expression levels of *asbt* in the Con group were significantly lower than those in all UDCA treatment groups, but significantly higher than those in the CDCA600 and CDCA1200 groups (*p* < 0.05). The expression levels of *fabp6a* in the Con group were significantly lower than those in the CDCA1200 group, but significantly higher than those in CDCA600, UDCA300, and UDCA1200 groups (*p* < 0.05). Finally, the expression levels of *fabp6b* in the Con group were significantly lower than those in the CDCA600 and UDCA300 groups (*p* < 0.05).

### 3.9. Correlation Analysis

**Figure 8:**
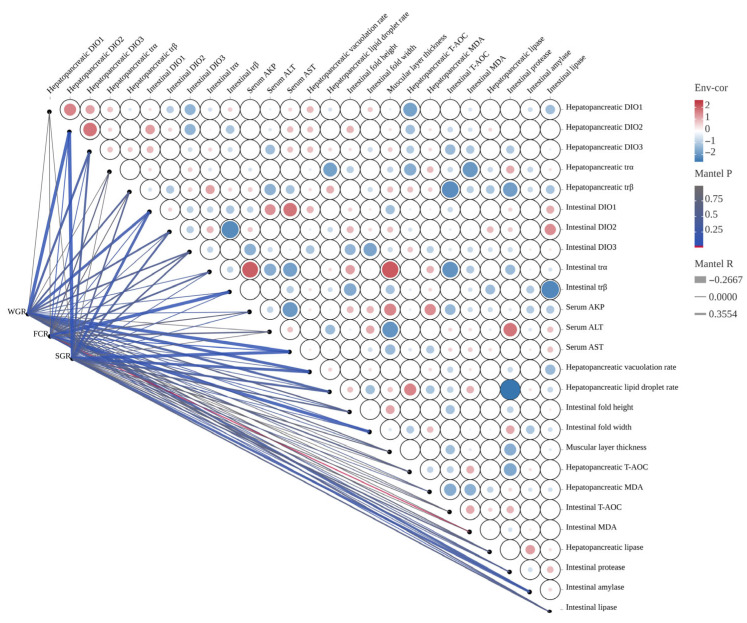
The Mantel correlation analysis between growth performance and key biochemical indices. The width of the edge corresponds to Mantel’s r value, and the color of the edge indicates statistical significance. Pairwise correlations between these variables are displayed using color gradients and circle areas to represent Pearson’s correlation coefficient.

The Mantel correlation analysis between growth performance and key biochemical indices is presented in Figure 8. WGR exhibited a significant negative correlation with hepatopancreatic DIO2, intestinal DIO1, serum AST, hepatopancreatic vacuolation rate, and intestinal α-amylase (R > −0.2667, *p* < 0.05). FCR showed a significant positive correlation with hepatopancreatic DIO2, serum AST, and intestinal MDA (R < 0.3554, *p* < 0.05). However, it was significantly negatively correlated with intestinal trβ and fold width (R > −0.2667, *p* < 0.05). Additionally, SGR demonstrated a significant negative correlation with hepatopancreatic DIO2, intestinal DIO1, serum AST, hepatopancreatic vacuolation rate, lipid droplet rate, and intestinal α-amylase (R > −0.2667, *p* < 0.05).

**Figure A2:**
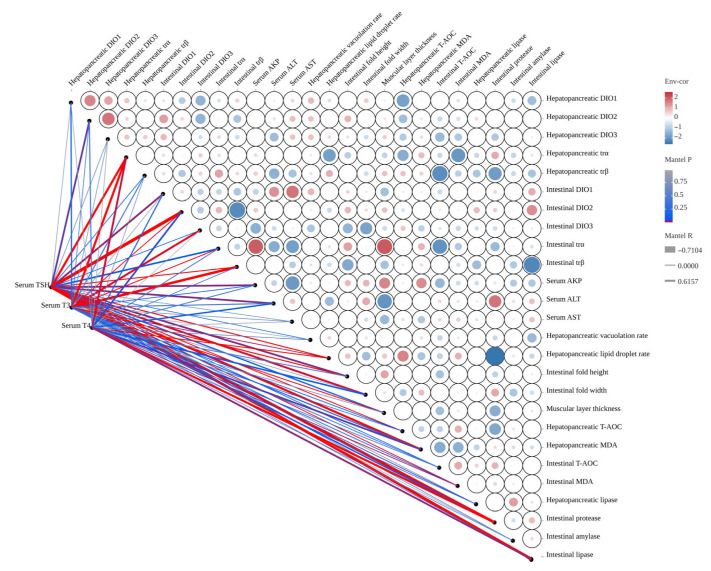
The Mantel correlation analysis for thyroid function and key biochemical indices. The width of the edge corresponds to Mantel’s r value, and the color of the edge indicates statistical significance. Pairwise correlations between these variables are displayed using color gradients and circle areas to represent Pearson’s correlation coefficient.

The Mantel correlation analysis for thyroid function and key biochemical indices is presented in Figure A2. Serum TSH was significantly negatively correlated with hepatopancreatic DIO2 and *trα*, intestinal DIO1, intestinal DIO2 and *trα*, serum AKP and ALT, hepatopancreatic vacuolation rate, intestinal fold height and width, mucosa layer thickness, hepatopancreatic MDA, and intestinal protease and lipase (R > −0.7104, *p* < 0.05). In contrast, serum TSH was significantly positively correlated with hepatopancreatic trβ, intestinal DIO3 and *trβ*, hepatopancreatic lipid droplet rate, and both hepatopancreatic T-AOC and MDA (R < 0.6157, *p* < 0.05). Serum T3 was significantly negatively correlated with hepatopancreatic DIO1 and *trα*, intestinal DIO3 and *trβ*, and intestinal protease (R > −0.7104, *p* < 0.05). However, it was significantly positively correlated with intestinal DIO2 and *trα*, serum AKP, hepatopancreatic lipid droplet rate, intestinal fold height, mucosa layer thickness, hepatopancreatic T-AOC and lipase, and intestinal MDA (R < 0.6157, *p* < 0.05). Serum T4 was significantly negatively correlated with hepatopancreatic trα, intestinal DIO1 and DIO2, serum ALT, intestinal fold width, hepatopancreatic MDA and lipase, and intestinal protease and lipase (R > −0.7104, *p* < 0.05). On the other hand, serum T4 was significantly positively correlated with hepatopancreatic lipid droplet rate and T-AOC, as well as intestinal MDA (R < 0.6157, *p* < 0.05).

## 4. Discussion

Existed studies have aimed to estimate the optimal types and levels of BAs in the diets of various fish species [9]. Notably, synergistic effects between BAs and balanced nutrient profiles have been documented, highlighting that the growth-promoting effects of CDCA and UDCA in fish are highly context-dependent, primarily modulated by dietary lipid/carbohydrate ratios and dosage [17,20]. For instance, supplementation with 300 and 900 mg/kg CDCA in a 6% soybean oil-based diet (lipid content: 12%) significantly enhanced growth performance in *L. crocea* [14]. In contrast, identical CDCA in a 12% fish oil-based diet (lipid content: 18%) failed to improve growth performance [16]. Additionally, 900 mg/kg CDCA supplementation mitigated growth retardation induced by a high-lipid (15.96%) diet in yellow catfish *Pelteobagrus fulvidraco* [25]. However, no significant growth improvements were observed in *M. salmoide* fed high-lipid (lipid content: 18.08%) diets supplemented with 300, 600, or 900 mg/kg CDCA [17]. Furthermore, dietary supplementation with 1000 mg/kg CDCA alleviated growth retardation induced by a high-carbohydrate (10% α-starch) diet in *M. salmoide* [26]. On the other hand, supplementation with 50 and 100 mg/kg UDCA in a diet (lipid content: 11–12%) significantly improved growth performance in *L. crocea* [20]. Similarly, 250 mg/kg UDCA supplementation in a 10% squid liver oil-based diet (lipid content: 12%) significantly enhanced growth performance in juvenile red sea bream (*Pagrus major*) [27], whereas no significant growth differences were observed in Japanese flounder (*Paralichthys olivaceus*) fed a 5% pollack oil-based diet (lipid content: 12%) supplemented with 250 mg/kg CDCA [19]. A previous study indicated that a dietary protein/lipid ratio of 47/12 was optimal for *L. polyactis* [6]. In the current study, we observed that dietary supplementation with CDCA and UDCA (300 and 600 mg/kg) mitigated growth retardation induced by a suboptimal protein-to-lipid ratio (48/6) diet in *L. polyactis*. This finding aligns with Yin’s perspective, which suggests that an imbalance between energy supply and growth needs could lead to metabolic disruptions in fish, resulting in impaired nutrient utilization [17]. The growth-promoting effect of BAs in *L. polyactis* may therefore be attributed to their multiple functions in regulating nutrients absorption, lipid utilization, and energy metabolism.

Previous studies have demonstrated that dietary BAs enhance feed utilization and increase total lipase activity in the liver and intestine of fish [15,28]. Similar findings were reported regarding the effects of dietary UDCA on total lipase activity in juvenile *L. crocea* [20]. Diversely, dietary CDCA did not significantly influence total lipase activity in the liver of *L. crocea* fed a high-lipid diet or in *M. salmoide* fed a 6% soybean oil diet [14,17]. Consequently, based on our results in *L. polyactis*, the growth-promoting effects of exogenous BAs may be linked to enhanced utilization of protein and lipids, as well as the increased activities of digestive enzymes in the intestine. However, further investigation is needed to elucidate the correlation between BA concentration and enzyme activities.

The synthesis and secretion of THs are stimulated by TSH in thyroid follicular cells [29]. In turn, free THs in circulation exert inhibitory effects on TSH secretion through the coordinated negative feedback regulation of the hypothalamic-pituitary-thyroid (HPT) axis and TH binding proteins [30]. Our results showed that dietary supplementation with CDCA and UDCA significantly altered the levels of TSH and TH in *L. polyactis*, suggesting that both CDCA and UDCA treatments reduced TH synthesis. Notably, a significant reduction in the levels of FT3 and FT4 were observed in the UDCA1200 group, indicating that only supplementation with 1200 mg/kg UDCA effectively lowered circulating free TH levels in *L. polyactis*. In peripheral tissues, deiodinases DIO1 and DIO2 are responsible for outer-ring deiodination (ORD), which converts T4 to T3, as well as metabolizing reverse triiodothyronine (rT3) to 3, 3′-diiodo-L-thyronine (T2) [29]. Conversely, DIO3 mediates inner-ring deiodination (IRD), inactivating T3 by converting it to T2 and modifying T4 into rT3, a biologically inactive form [30]. Previous studies have established that BAs are critically dependent on the BAs–TGR5–cAMP–DIO2 signaling pathway in the regulation of energy homeostasis [31]. Therefore, this pathway of BAs–TGR5–cAMP–DIO2 can induce local T3 production and metabolism [32]. However, limited research has been conducted on the effects of dietary BA supplementation on TH metabolism. In *L. polyactis*, our results revealed a dose-dependent relationship between the concentration of CDCA or UDCA in diets and the processes of ORD and IRD in the hepatopancreas and intestine. For example, supplementation with 300 mg/kg CDCA inhibited both ORD and IRD in the hepatopancreas, while supplementation with 600 mg/kg and 1200 mg/kg CDCA promoted hepatopancreatic ORD and IRD, respectively. In contrast, supplementation with 300 mg/kg UDCA promoted hepatopancreatic IRD, whereas 600 mg/kg and 1200 mg/kg UDCA supplementation both enhanced the process of hepatopancreatic ORD and IRD.

Tissue damage serves as a critical indicator for evaluating overall health. Research has demonstrated that the type and dosage of dietary BAs can differentially affect hepatopancreatic and intestinal lipid accumulation, as well as the overall health status of fish [17,33]. Previous studies have shown that dietary supplementation with CDCA significantly reduces hepatic lipid droplet content induced by a high-fat diet in juvenile largemouth bass (*Micropterus salmoides*), with the most pronounced effect occurring at a supplementation level of 900 mg/kg CDCA [17,25]. Conversely, supplementation with 100 mg/kg CDCA significantly increased the size of hepatic lipid droplets in grass carp [33]. Furthermore, supplementation with 600 mg/kg and 900 mg/kg CDCA notably mitigated the adverse effects of high-fat diets on intestinal fold height in juvenile *M. salmoides* [17]. In contrast, dietary supplementation with 400 mg/kg and 800 mg/kg CDCA significantly alleviated damage to the intestinal mucosal folds in juvenile white shrimp (*Litopenaeus vannamei*) fed a low fishmeal diet, though the effects varied with the different CDCA supplementation levels [34]. While the effects of CDCA supplementation have been extensively studied, fewer studies have focused on the impacts of dietary UDCA on hepatopancreatic and intestinal histology [20]. In the present study, an unsatisfactory protein/lipid ratio (48/6) diet resulted in hepatopancreatic cells vacuolation and lipid accumulation in the hepatopancreas of *L. polyactis*. Supplementation with 300 mg/kg CDCA and 600 mg/kg UDCA effectively reduced the vacuolation rate and lipid droplet content in the hepatopancreas. Additionally, the morphology of intestinal mucosal folds in *L. polyactis* fed the unsatisfactory protein/lipid ratio (48/6) diet was improved by the supplementation of 300 mg/kg UDCA. These findings suggest that specific types and concentrations of BAs can enhance hepatic and intestinal health in *L. polyactis*, although the underlying regulatory mechanisms remain unclear.

In addition to histological examination, serum levels of ALT, AST and ALP are key biomarkers for assessing cellular damage in the liver [35]. ALT is considered more specific for liver injury than AST [36]. Moreover, ALP plays a crucial role in cellular regeneration and wound healing when fish are subjected to stress or injury [37]. In this study, dietary supplementation with 1200 mg/kg CDCA appeared to attenuate hepatopancreatic damage, which was inconsistent with the histological analysis results. In contrast, previous research indicated that a high-starch diet supplemented with BAs resulted in lower ALT, AST and ALP activities in plasma, as well as a reduced vacuolization rate in liver of *M. salmoides* [35]. Given the findings of the previous report [35], these discrepancies may be attributed to variations in fish species, types and concentrations of BAs, and the nutrient composition of the diet.

The oxidative stress response can interfere with hepato-intestinal lipid metabolism and immune functions by modulating BA homeostasis in fish [9]. The increase in MDA production serves as a marker of lipid peroxidation, while T-AOC, SOD, GSH, and CAT levels reflect the ability to scavenge excessive superoxide radicals [17,25]. In the present study, dietary supplementation with CDCA and UDCA resulted in an increase in hepatopancreatic MDA content and a decrease in intestinal MDA content in *L. polyactis* fed a diet with an unsatisfactory protein/lipid ratio (48/6). However, previous studies showed that higher hepatic MDA content was observed in *M. salmoides* fed high-fat and high- starch diets, and dietary CDCA supplementation alleviated oxidative damage [17,35]. Hence, these findings suggest that while CDCA and UDCA may improve intestinal antioxidant capacity, they can induce oxidative damage in the hepatopancreas of *L. polyactis* when fed an unsatisfactory protein/lipid ratio (48/6) diet. Additionally, classic antioxidant enzymes (e.g., SOD and CAT) and non-enzymatic antioxidant components (e.g., GSH) play critical roles in protecting cells and tissues from oxidative damage [38]. Supplementation with 900 mg/kg CDCA significantly increased the expression levels of sod, though various levels of CDCA had no significant effects on the expression levels of *gsh-px* in liver of *M. salmoides* fed a high-fat diet [17]. In contrast, our results indicated that the different levels of CDCA and UDCA supplementation in experimental diets led to varied adaptive responses in the hepato-intestinal antioxidative defense systems of *L. polyactis*. These responses were characterized by dynamic alterations in the activities of SOD and CAT activities, as well as changes in GSH content.

TNFα, IL1β, and IL-6 are pivotal mediators in the inflammatory response, with both IL1β and IL-6 acting as pro-inflammatory cytokines [39]. TNFα plays a key role in various cellular processes that maintain intestinal integrity and regulate the pathogenesis of intestinal inflammation [40]. Previous studies have demonstrated that the expression levels of il-1β and il-6 in the intestine were elevated in fish fed high soybean oil diets compared to those fed fish oil diets, and that CDCA supplementation alleviated intestinal inflammation in *L. crocea* [39]. Similarly, certain levels of UDCA supplementation significantly reduced the expression levels of il-1β in *L. crocea* [21]. In contrast, compared to experimental diets without the supplementation, only experimental diets contained fermented BAs at a concentration of 0.05% significantly down-regulated TNFα and IL1β expression in the spleen of *M. salmoide*, while other levels of fermented BAs led to significant up-regulation of these cytokines [41]. In the present study, dietary supplementation with both CDCA and UDCA significantly up-regulated the expressions of TNFα, IL1β, and IL-6, suggesting that both BAs contributed to intestinal inflammation in *L. polyactis*. In rat, UDCA exacerbated indomethacin-induced small intestinal inflammation, but ameliorated inflammation in inflammatory bowel disease [42,43]. We hypothesize that the pathogenic mechanisms may influence the effectiveness of UDCA in modulating inflammation. Therefore, the investigation of factors contributing to the exacerbation of intestinal inflammation should also consider the impact of dietary components in *L. polyactis*.

The activation of FXR and TGR5 plays a crucial role in the enterohepatic circulation of BAs [9]. The FXR-RXRα heterodimer regulates the transcription of target genes involved in BA metabolism, whereas PPAR, TR, and FXR engage in competitive binding to RXRα, leading to mutual suppression of transcriptional activity [9,44,45]. In the liver, CYP7A1 and CYP27α regulate the classic and alternative pathways of BA synthesis, respectively [8]. Our study indicated that dietary supplementation with CDCA and UDCA both promote the classic pathway of BA synthesis and energy metabolism in hepatopancreas of *L. polyactis* fed a diet with an unsatisfactory protein/lipid ratio (48/6) diet. However, only low levels of BA in the diet enhanced hepatopancreatic BA metabolism via increasing the expression levels of *fxr* and *tgr5*. Conversely, dietary supplementation with 900 mg/kg CDCA was the only treatment that promoted the alternative pathway of BA synthesis by up-regulating the expression levels of *cyp27α* in the liver of *M. salmoides* fed a high-lipid diet [17]. Interestingly, similar levels of CDCA supplementation activated Fxr to inhibit both BA synthesis pathways in the liver of *P. fulvidraco* fed a high-lipid diet, as indicated by up-regulated expression levels of *fxr* and down-regulated expression levels of *cyp7α1* and *cyp27α1* [25,46]. Moreover, UDCA supplementation has been shown to enhance lipid synthesis, transport, and the classic pathway of BA synthesis in the hepatocytes of juvenile *L. crocea* [20]. UDCA supplementation also significantly influenced the expression of genes involved in intestinal lipid metabolism in *L. crocea* [20]. In the present study, CDCA supplementation at 600–1200 mg/kg and UDCA supplementation at 300–600 mg/kg were associated with enhanced energy metabolism and BAs transport, as indicated by the regulation of genes involved in intestinal BA and TR metabolic signaling pathways. CDCA was found to be a more effective FXR agonist than UDCA [9], with our results confirming that CDCA was a more potent Fxr and Tgr5 agonists in the intestine of *L. polyactis*. In addition, CDCA supplement could inhibit the absorption of BAs in the intestine, while UDCA supplement could promote the transports of BAs through the intestine. These findings suggest that the optimal dose of BAs can significantly affect hepatic–intestinal metabolic processes in fish, though the effective dosage may vary depending on the fish species and the nutritional composition of the diet. Considering the farming costs and effectiveness, dietary supplementation with 300 mg/kg CDCA can alleviate hepatopancreatic injury, and dietary supplementation with 300 mg/kg UDCA can improve the intestinal morphology in *L. polyactis* fed a high protein–lipid ratio feed.

Dietary nutrients regulate metabolic processes across various tissues following digestion and absorption [47,48]. Previous studies have suggested that the mechanism by which dietary BA supplementation promotes growth may involve enhanced nutrients utilization and deposition, reduced stress responses, and improved liver function in fish [25,35]. In certain fish species, components of the thyroid axis also influence the growth hormone (GH)/insulin-like growth factor I (IGF-I) axis, indicating interactions between thyroid and growth regulatory axes [49]. In this study, WGR and SGR, key indicators of growth performance, were negatively correlated with hepatic–intestinal ORD, liver injury, and amylase utilization. These findings suggest that dietary supplementation with CDCA or UDCA promotes growth via modulating hepatic–intestinal energy metabolism and mitigating liver damage. Plasma homeostasis of T3 and T4 in vertebrates is maintained by the hypothalamic–pituitary–thyroid (HPT) axis and TR signaling pathway, with TSH secretion regulated via T4-mediated feedback mechanism through differential degradation by DIO2 [50]. BAs participate in the TGR5–cAMP–DIO2 pathway, thereby modulating local THs metabolism [32]. However, the effects of dietary BAs supplementation on interaction between HPT axis and TR signaling pathway remain incompletely understood. In the present study, correlation analysis revealed significant positive relationships between hepatic–intestinal ORD, hepatic–intestinal trα transcripts, and serum TSH, as well as significant negative correlations between hepatic–intestinal IRD, hepatic–intestinal *trβ* transcripts, and serum TSH. These results suggest that dietary supplementation with CDCA or UDCA may up-regulate TSH, thereby activating hepatic–intestinal ORD and T3-trα signaling pathway. Furthermore, thyroid dysfunction can contribute to liver function disorders [51]. Consistently, our findings also revealed significant positive correlations between serum T3 and AKP, as well as serum T4 and ALT, highlighting the close interplay between thyroid function and hepatic health.

## 5. Conclusions

Commercial feeds with a higher protein-to-lipid ratio, when used in intensive farming conditions for *L. polyactis*, often lead to tissue damage, primarily due to inadequate nutrient intake. The present results revealed that supplementation of CDCA or UDCA at concentrations ranging from 300 to 1200 mg/kg could improve growth performance and modulated TH metabolism of *L. polyactis* fed common commercial feeds. The supplementation of both BAs at varying concentrations resulted in distinct patterns of deiodination in the hepatopancreas and intestine. Dietary supplementation of both BAs enhanced hepatopancreatic energy metabolism and BA synthesis via the TH signaling pathway, while also bolstering intestinal antioxidant capacity. However, these benefits were accompanied by increased intestinal inflammation and hepatopancreatic oxidative stress. Notably, supplementation with 300 mg/kg CDCA and 600 mg/kg UDCA effectively reduced the vacuolation rate and lipid droplet content in the hepatopancreas, correlating with enhanced digestion of proteins and lipids. Furthermore, the supplementation of 300 mg/kg UDCA improved the morphology of intestinal mucosal folds. These findings suggest that dietary supplementation with CDCA and UDCA can, respectively, improve the hepatopancreatic and intestinal health of *L. polyactis* when fed a high protein–lipid ratio feed. The optimal supplemental level of CDCA or UDCA is 300 mg kg^−1^. In addition, our results underscore that different concentrations and types of BAs in the diet lead to distinct metabolic pathways in the hepatopancreas and intestine. Further investigations are required to fully elucidate the underlying mechanisms involved in these pathways, which will provide a deeper understanding of the role of BAs in the nutritional responses of *L. polyactis* to various dietary components.

## Figures and Tables

**Table 1:** Proximate composition of the experimental diets.

Ingredients%	Con	CDCA300	CDCA600	CDCA1200	UDCA300	UDCA600	UDCA1200
Crude protein	47.55	47.55	47.71	48.04	47.68	47.67	48.17
Crude lipid	5.90	6.00	5.90	5.90	5.90	5.90	5.90
Ash	11.60	12.00	12.00	11.70	12.00	11.70	12.20

**Table 2:** Key parameters of the commercial kits.

Assay Item	Substrate	Reaction System/μL	Label	Wavelength/nm	Assay Method	Enzyme Unit	Kit Catalog Number
DIO1	TMB	150	HRP	450	Sandwich ELISA	ng/g tissue	ml090524
DIO2	TMB	150	HRP	450	Sandwich ELISA	ng/g tissue	ml100959
DIO3	TMB	150	HRP	450	Sandwich ELISA	ng/g tissue	ml100958
T-SOD	Xanthine, WST-8	200	/	450	WST-8 method	U/g tissue	ml076328
CAT	H_2_O_2_, MoO_4_^2−^	80	/	405	Colorimetric method	μmol/min/g tissue	ml092622
T-AOC	ABTS, ABTS^+^	200	/	734	ABTS method	μmol Trolox/g tissue	ml076332
GSH	DTNB, GSH	200	/	412	Colorimetric method	μmol/g tissue	ml076450
MDA	MDA, TBA	400	/	532, 600	TBA method	nmol/g tissue	ml022446
IL-8	TMB	150	HRP	450	Sandwich ELISA	ng/L	ml028580
IL-1β	TMB	150	HRP	450	Sandwich ELISA	ng/L	ml003463
TNF-α	TMB	150	HRP	450	Sandwich ELISA	ng/L	ml002095
Total protease	TMB	150	HRP	450	Sandwich ELISA	U/g tissue	ml063790
Total lipase	TMB	150	HRP	450	Sandwich ELISA	U/g tissue	ml036371
Total amylase	Starch, DNS	300	/	540	DNS method	U/g tissue	ml076677

**Table 3:** Primer sequences for qRT-PCR analysis.

Target Genes	Primer Sequences (5′–3′)	Tm (°C)	GenBank Accession No.
*18s-qF*	GTGGAGGCATGGTGGTGGATTAC	55.7	MT330379.1
*18s-qR*	GGACCTGGTTGGAAACAGCTCTG	55.3	
*asbt-qF*	ATCGCTGTGGTTGGAGGAAT	60.8	WMHY01000022.1
*asbt-qR*	GCTGGAAGATGCTGTAGATGAG	60.8	
*fabp6a-qF*	ATGCCAAGACGGACCACAA	56.6	JAFMOB010000055.1
*fabp6a-qR*	GCTCACATTCCTCACCAACG	55.3	
*fabp6b-qF*	TGCTTGGTATCCCTGATGACAT	57.2	JAFMOB010000192.1
*fabp6b-qR*	TCCTTGCCGATGGTGAACTT	56.6	
*ost-α-qF1*	ACCTCTGCCTGCTACTTTGC	55.7	WMHY01006574.1
*ost-α-qR1*	TGAGCAGGAAGAGAGAACGC	56.7	
*fxr-qF*	TGGTGGTGGCTGTGAGATG	58.0	JAFMOB010000007.1
*fxr-qR*	CCTCCTGCTGTCTGTGTTCT	57.3	
*t* *gr* *5* *-qF*	GCTCCACCACTCACATCGT	57.9	JAFMOB010000023.1
*t* *gr* *5* *-qR*	TCACACTGCCACCTCCTCT	57.1	
*ppar* *α* *-qF*	ATGACTACAGCAGTGATGAC	57.8	JAFMOB010000073.1
*ppar* *α* *-qR*	GGAGAACAAGAAGATGAAGATG	58.7	
*trβ-qF*	CCGTCATCTCATTCTGGCCT	51.4	JAFMOB010000022.1
*trβ-qR*	GAAGCGGGAGGGAAGAGTG	50.4	
*trα-qF*	TCCTGTTGCTGGTGTGTTGT	59.5	JAFMOB010000009.1
*trα-qR*	CCTGATGAAGGTGACGGACC	59.8	
*cyp7a1-qF*	GCCACACCACAGAGAACCT	57.1	JAFMOB010000591.1
*cyp7a1-qR*	GCGAGCCTGACACTTATCTTC	58.4	
*cyp27a1-qF*	TCTATGGTCCTCTGTGGAAGTC	57.8	WMHY01082556.1
*cyp27a1-qR*	TTCGTTGATGGTGTTAGCGTAT	55.8	

**Table 4:** Effects of dietary different BAs on the growth performance of *L. polyactis*.

Groups	WGR (%)	CF (g/cm^3^)	FCR	SGR
Con	104.95 ± 5.89 ^b^	1.67 ± 0.03	1.90 ± 0.10 ^a^	1.16 ± 0.05 ^b^
CDCA300	133.61 ± 7.05 ^a^	1.60 ± 0.04	1.70 ± 0.08 ^ab^	1.38 ± 0.05 ^a^
CDCA600	135.75 ± 6.23 ^a^	1.65 ± 0.03	1.64 ± 0.07 ^b^	1.40 ± 0.04 ^a^
CDCA1200	121.81 ± 4.62 ^a^	1.69 ± 0.04	1.73 ± 0.06 ^ab^	1.31 ± 0.03 ^a^
UDCA300	127.44 ± 6.40 ^a^	1.63 ± 0.03	1.73 ± 0.08 ^ab^	1.34 ± 0.05 ^a^
UDCA600	133.40 ± 5.85 ^a^	1.62 ± 0.03	1.82 ± 0.07 ^ab^	1.39 ± 0.04 ^a^
UDCA1200	121.22 ± 5.37 ^a^	1.62 ± 0.04	1.91 ± 0.07 ^a^	1.30 ± 0.04 ^a^

Note: The results are presented as the means ± SE (*n* = 45). Values with different superscripts in the same column are significantly different (*p* < 0.05).

**Table 5:** Effects of dietary BA supplementation on hepatopancreatic antioxidant parameters in *L. polyactis*.

Parameters	Con	CDCA300	CDCA600	CDCA1200	UDCA300	UDCA600	UDCA1200
T-AOC (μmol Trolox/g tissue)	4.49 ± 0.12 ^b^	5.59 ± 0.29 ^a^	2.08 ± 0.33 ^d^	4.91 ± 0.18 ^b^	4.36 ± 0.15 ^b^	2.23 ± 0.16 ^d^	3.74 ± 0.18 ^c^
T-SOD (U/g tissue)	560.21 ± 4.38 ^b^	2001.87 ± 16.02 ^a^	571.99 ± 2.10 ^b^	335.33 ± 2.60 ^d^	258.89 ± 3.07 ^f^	525.20 ± 7.01 ^c^	294.57 ± 2.01 ^e^
CAT (μmol/min/g tissue)	87.35 ± 1.37 ^b^	86.71 ± 0.80 ^b^	82.12 ± 0.53 ^d^	84.13 ± 0.66 ^cd^	85.42 ± 0.39 ^bc^	86.76 ± 0.44 ^b^	91.69 ± 0.35 ^a^
MDA (nmol/g tissue)	5.00 ± 0.15 ^d^	6.03 ± 0.42 ^c^	7.32 ± 0.22 ^a^	6.33 ± 0.12 ^bc^	7.37 ± 0.19 ^a^	6.18 ± 0.07 ^c^	6.91 ± 0.16 ^ab^
GSH (μmol/g tissue)	19.49 ± 0.26 ^b^	11.44 ± 0.23 ^f^	12.45 ± 0.27 ^e^	18.51 ± 0.43 ^cd^	19.05 ± 0.19 ^bc^	20.36 ± 0.34 ^a^	17.93 ± 0.11 ^d^

Note: The values were expressed as mean ± SE (*n* = 3), and different little letters indicate significant differences among all the groups (*p* < 0.05).

**Table 6:** Effects of dietary BA supplementation on hepatopancreatic injury biomarkers in *L. polyactis*.

Parameters	Con	CDCA300	CDCA600	CDCA1200	UDCA300	UDCA600	UDCA1200
ALP (nmol/min/mL)	40 ± 0.01 ^cd^	30 ± 0.01 ^cd^	120 ± 0.01 ^b^	30 ± 0.01 ^cd^	190 ± 0.01 ^a^	10 ± 0.01 ^d^	40 ± 0.02 ^c^
ALT (nmol/min/mL)	57.91 ± 0.36 ^c^	65.6 ± 0.37 ^ab^	63.77 ± 0.23 ^b^	56.73 ± 0.43 ^c^	54.17 ± 1.09 ^d^	56.97 ± 0.36 ^c^	66.89 ± 1.17 ^a^
AST (nmol/min/mL)	97.58 ± 0.91 ^b^	92.95 ± 0.75 ^cd^	91.29 ± 0.75 ^d^	94.73 ± 0.69 ^c^	88.26 ± 1.02 ^e^	97.71 ± 0.98 ^b^	102.73 ± 0.37 ^a^

Note: The values were expressed as mean ± SE (*n* = 3), and different little letters indicate significant differences among all the groups (*p* < 0.05).

**Table 7:** Effects of dietary BA supplementation on intestinal antioxidant parameters in *L. polyactis*.

Parameters	Con	CDCA300	CDCA600	CDCA1200	UDCA300	UDCA600	UDCA1200
T-AOC (μmol Trolox/g tissue)	18.1 ± 0.12 ^a^	15.14 ± 0.09 ^d^	16.75 ± 0.07 ^b^	13.54 ± 0.08 ^e^	11.37 ± 0.1 ^f^	15.5 ± 0.21 ^c^	13.82 ± 0.15 ^e^
T-SOD (U/g tissue)	124.59 ± 3.98 ^d^	124.69 ± 1.05 ^d^	211.9 ± 5.03 ^b^	272.74 ± 22.96 ^a^	211.5 ± 6.19 ^b^	127.39 ± 2.35 ^d^	154.87 ± 3.84 ^c^
CAT (μmol/min/g tissue)	38.19 ± 0.18 ^e^	73.79 ± 0.13 ^c^	40.28 ± 0.17 ^d^	77.01 ± 0.22 ^b^	78.47 ± 0.13 ^a^	13.14 ± 0.15 ^f^	77.38 ± 0.18 ^b^
MDA (nmol/g tissue)	10.27 ± 0.96 ^a^	7.95 ± 0.38 ^b^	6.53 ± 0.14 ^c^	6.91 ± 0.13 ^bc^	5.96 ± 0.22 ^cd^	5.18 ± 0.16 ^d^	7.96 ± 0.29 ^b^
GSH (μmol/g tissue)	1.06 ± 0.01 ^b^	0.96 ± 0.01 ^c^	1.25 ± 0.01 ^a^	0.98 ± 0.01 ^c^	0.2 ± 0.01 ^f^	0.85 ± 0.01 ^d^	0.72 ± 0.01 ^e^

Note: The values were expressed as mean ± SE (*n* = 3), and different little letters indicate significant differences among all the groups (*p* < 0.05).

**Table 8:** Effects of dietary BA supplementation on the pro-inflammatory cytokines in the intestine of *L. polyactis*.

Parameters	Con	CDCA300	CDCA600	CDCA1200	UDCA300	UDCA600	UDCA1200
IL-8 (ng/L)	26.24 ± 3.23 ^d^	125.54 ± 3.47 ^b^	123.23 ± 3.29 ^b^	138.84 ± 0.88 ^a^	62.95 ± 2.88 ^c^	122.37 ± 4.74 ^b^	63.97 ± 3.99 ^c^
IL-1β (ng/L)	71.94 ± 3.52 ^e^	149.73 ± 3.45 ^c^	185.31 ± 3.41 ^b^	202.56 ± 3.17 ^a^	98.07 ± 0.34 ^d^	184.76 ± 2.69 ^b^	100.56 ± 4.49 ^d^
TNF-α (ng/L)	83.85 ± 2.86 ^e^	138.65 ± 1.86 ^c^	150.67 ± 2.33 ^b^	170.13 ± 3.13 ^a^	94.69 ± 2.29 ^d^	165.46 ± 1.6 ^a^	137.3 ± 1.8 ^c^

Note: The values were expressed as mean ± SE (*n* = 3), and different little letters indicate significant differences among all the groups (*p* < 0.05).

## Data Availability

The original contributions presented in this study are included in the article. Further inquiries can be directed to the corresponding authors.

## Appendix A

(A) Relationship between dietary addition CDCA levels and WGR. The regression equations are WGR = 0.1019CDCAc + 105.0(CDCAc = 323.68, R^2^ = 0.98979), WGR = −0.01859CDCAc + 144.0(CDCAc = 323.68, R^2^ = 0.8286).
(B) Relationship between CDCA levels and CF in dietary addition. The regression equations are CF = −0.0004111CDCAc + 1.713(CDCAc = 286.96, R^2^ = 0.9716), CF = 0.0001151CDCAc + 1.562(CDCAc = 286.96, R^2^ = 0.9505).
(C) Relationship between CDCA levels and FCR in dietary supplementation. The regression equations are FCR = −0.0005278CDCAc +1.915(CDCAc = 588.24, R^2^ = 0.9460), FCR = 0.0001556CDCAc + 1.1513(CDCAc = 588.24, R^2^ = 0.6975).
(D) Relationship between dietary CDCA addition level and SGR. The regression equations are SGR = 0.0009222CDCAc + 1.180(CDCAc = 282.21, R^2^ = 0.9874), SGR = −0.0001302CDCAc + 1.477(CDCAc = 282.21, R^2^ = 0.7406).
(E) Relationship between UDCA levels in diet addition and WGR. The regression equations are WGR = 0.04458UDCAc + 104.8(UDCAc = 597.90, R^2^ = 0.9725), WGR = −0.02115UDCAc + 144.1(UDCAc = 597.90, R^2^ = 0.8286).
(F) Relationship between UDCA levels and CF in dietary supplementation. The regression equations are CF = −0.0007222UDCAc + 1.707(UDCAc = 290.76, R^2^ = 0.9531), CF = 0.0002683UDCAc + 1.420(CDCAc = 290.76, R^2^ = 0.9733).
(G) Relationship between UDCA levels and FCR in dietary supplementation. The regression equations are FCR = −0.0004889UDCAc+ 1.923(UDCAc = 294.26, R^2^ = 0.9013), FCR = 0.0001500UDCAc+ 1.735(UDCAc = 294.26, R^2^ = 0.8750).
(H) Relationship between UDCA level and SGR in dietary addition. The regression equations are SGR = 0.0003444UDCAc + 1.193(UDCAc = 574.43, R^2^ = 0.9366), SGR = −0.0001500UDCAc + 1.477(UDCAc = 574.43, R^2^ = 0.9580).
